# (*E*)-2-[(3,5-Di-*tert*-butyl-2-hydroxy­benzyl­idene)amino]benzonitrile

**DOI:** 10.1107/S1600536809019527

**Published:** 2009-05-29

**Authors:** Jian-Cheng Zhou, Nai-Xu Li, Chuan-Ming Zhang, Zheng-Yun Zhang

**Affiliations:** a College of Chemistry and Chemical Engineering, Southeast University, Nanjing 211189, People’s Republic of China

## Abstract

The asymmetric unit of the title compound, C_22_H_26_N_2_O, contains three crystallographically independent mol­ecules, in which the aromatic rings are oriented at dihedral angles of 21.74 (5), 27.59 (5) and 27.87 (5)°. Intra­molecular O—H⋯N hydrogen bonds result in the formation of planar six-membered rings, and these are nearly coplanar with the adjacent rings. In the crystal structure, π–π contacts between the benzene rings [centroid–centroid distances = 3.989 (2), 3.802 (1) and 3.882 (1) Å] may stabilize the structure.

## Related literature

For general background, see: Chen *et al.* (2008 ▶); Dao *et al.* (2000 ▶); Sriram *et al.* (2006 ▶); Weber *et al.* (2007 ▶). For bond-length data, see: Allen *et al.* (1987 ▶).
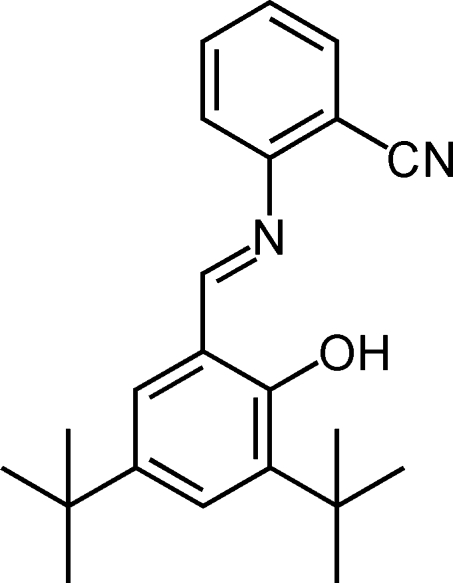


## Experimental

### 

#### Crystal data

C_22_H_26_N_2_O
*M* *_r_* = 334.45
Monoclinic, 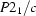

*a* = 27.9710 (4) Å
*b* = 7.32780 (11) Å
*c* = 29.7840 (4) Å
β = 104.5330 (2)°
*V* = 5909.37 (15) Å^3^
*Z* = 12
Mo *K*α radiation
μ = 0.07 mm^−1^
*T* = 294 K
0.30 × 0.20 × 0.20 mm

#### Data collection

Bruker SMART CCD area-detector diffractometer
Absorption correction: empirical (using intensity measurements) (*SADABS*; Bruker, 2000 ▶) *T*_min_ = 0.948, *T*_max_ = 0.986
30875 measured reflections
11513 independent reflections
4685 reflections with *I* > 2σ(*I*)
*R*_int_ = 0.130

#### Refinement

*R*[*F* ^2^ > 2σ(*F* ^2^)] = 0.061
*wR*(*F* ^2^) = 0.173
*S* = 0.81
11513 reflections
689 parameters
H atoms treated by a mixture of independent and constrained refinement
Δρ_max_ = 0.31 e Å^−3^
Δρ_min_ = −0.23 e Å^−3^

### 

Data collection: *SMART* (Bruker, 2000 ▶); cell refinement: *SAINT* (Bruker, 2000 ▶); data reduction: *SAINT*; program(s) used to solve structure: *SHELXS97* (Sheldrick, 2008 ▶); program(s) used to refine structure: *SHELXL97* (Sheldrick, 2008 ▶); molecular graphics: *SHELXTL* (Sheldrick, 2008 ▶); software used to prepare material for publication: *SHELXL97*.

## Supplementary Material

Crystal structure: contains datablocks I, global. DOI: 10.1107/S1600536809019527/hk2692sup1.cif

Structure factors: contains datablocks I. DOI: 10.1107/S1600536809019527/hk2692Isup2.hkl

Additional supplementary materials:  crystallographic information; 3D view; checkCIF report

## Figures and Tables

**Table 1:** Hydrogen-bond geometry (Å, °)

*D*—H⋯*A*	*D*—H	H⋯*A*	*D*⋯*A*	*D*—H⋯*A*
O3—H3*A*⋯N5	0.95 (2)	1.81 (2)	2.622 (2)	143 (2)
O2—H2*A*⋯N3	0.92 (3)	1.82 (3)	2.615 (2)	143 (2)
O1—H1*A*⋯N1	0.87 (2)	1.86 (2)	2.623 (2)	145 (2)

**Fig. 1.**
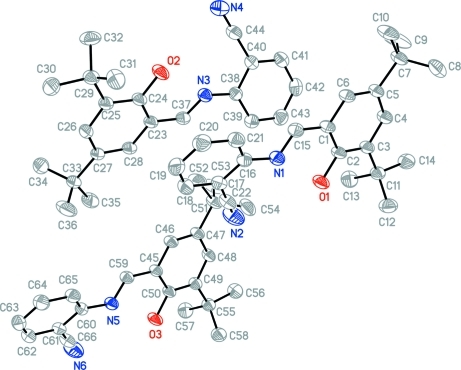
The molecular structure of the title molecule, with the atom-numbering scheme. Displacement ellipsoids are drawn at the 30% probability level. Hydrogen atoms have been omitted for clarity.

## supplementary crystallographic information

### Comment

Schiff base compounds have received considerable attention for many years, primarily due to various pharmacological activities, such as anticancer (Dao *et al.*, 2000) and anti-HIV (Sriram *et al.*, 2006). In addition, Schiff base compounds play important roles in the development of coordination chemistry related to magnetism (Weber, *et al.*, 2007) and catalysis (Chen, *et al.*, 2008). We report herein the crystal structure of the title compound.

The asymmetric unit of the title compound contains three crystallographicaly independent molecules (Fig. 1), in which the bond lengths (Allen *et al.*, 1987) and angles are within normal ranges. Rings A (C1-C6), B (C16-C21), C (C23-C28), D (C38-C43), E (C45-C50) and F (C60-C65) are, of course, planar and the dihedral angles between them are A/B = 21.74 (5), C/D = 27.59 (5) and E/F = 27.87 (5) °. Intramolecular O-H···N hydrogen bonds (Table 1) results in the formations of planar six-membered rings G (O1/N1/C1/C2/H1A), H (O2/N3/C23/C24/C37/H2A) and I (O3/N5/C45/C50/C59/H3A), in which they are oriented with respect to the adjacent rings at dihedral angles of A/G = 1.23 (5), C/H = 1.07 (5) and E/I = 1.30 (5) °.

In the crystal structure, the π–π contacts between the benzene rings, Cg1—Cg4^i^, Cg2—Cg3^i^ and Cg5—Cg6^ii^, [symmetry codes: (i) x, y - 1, z, (ii) 1 - x, 1/2 + y, 1/2 - z, where Cg1, Cg2, Cg3, Cg4, Cg5 and Cg6 are centroids of the rings A (C1-C6), B (C16-C21), C (C23-C28), D (C38-C43), E (C45-C50) and F (C60-C65), respectively] may stabilize the structure, with centroid-centroid distances of 3.989 (2), 3.802 (1) and 3.882 (1) Å, respectively.

### Experimental

For the preparation of the title compound, 3,5-di-*tert*-butyl-2 -hydroxybenzaldehyde (0.936 g, 4 mmol) and 2-aminobenzonitrile (0.531 g, 4.5 mmol) were dissolved in ethanol (25 ml). The mixture was heated to reflux for 6 h, and then cooled to room temperature. The solution was filtered and yellow crystals suitable for X-ray analysis formed after two weeks on slow evaporation of the solvent at room temperature.

### Refinement

H atoms (for OH) were located in a difference synthesis and refined isotropically. The remaining H atoms were positioned geometrically, with C-H = 0.93 and 0.96 Å for aromatic and methyl H, respectively, and constrained to ride on their parent atoms, with U_iso_(H) = xU_eq_(C), where x = 1.5 for methyl H and x = 1.2 for aromatic H atoms.

### Crystal data

**Table:**

C_22_H_26_N_2_O	*F*(000) = 2160
*M**_r_* = 334.45	*D*_x_ = 1.128 Mg m^−^^3^
Monoclinic, *P*2_1_/*c*	Mo *K*α radiation, λ = 0.71073 Å
Hall symbol: -P 2ybc	Cell parameters from 2369 reflections
*a* = 27.9710 (4) Å	θ = 3.1–27.0°
*b* = 7.32780 (11) Å	µ = 0.07 mm^−^^1^
*c* = 29.7840 (4) Å	*T* = 294 K
β = 104.5330 (2)°	Block, yellow
*V* = 5909.37 (15) Å^3^	0.30 × 0.20 × 0.20 mm
*Z* = 12	

### Data collection

**Table:**

Bruker SMART CCD area-detector diffractometer	11513 independent reflections
Radiation source: fine-focus sealed tube	4685 reflections with *I* > 2σ(*I*)
graphite	*R*_int_ = 0.130
φ and ω scans	θ_max_ = 26.0°, θ_min_ = 1.8°
Absorption correction: empirical (using intensity measurements) (*SADABS*; Bruker, 2000)	*h* = −32→34
*T*_min_ = 0.948, *T*_max_ = 0.986	*k* = −8→9
30875 measured reflections	*l* = −36→27

### Refinement

**Table:**

Refinement on *F*^2^	Secondary atom site location: difference Fourier map
Least-squares matrix: full	Hydrogen site location: inferred from neighbouring sites
*R*[*F*^2^ > 2σ(*F*^2^)] = 0.061	H atoms treated by a mixture of independent and constrained refinement
*wR*(*F*^2^) = 0.173	*w* = 1/[σ^2^(*F*_o_^2^) + (0.0618*P*)^2^] where *P* = (*F*_o_^2^ + 2*F*_c_^2^)/3
*S* = 0.81	(Δ/σ)_max_ < 0.001
11513 reflections	Δρ_max_ = 0.31 e Å^−^^3^
689 parameters	Δρ_min_ = −0.23 e Å^−^^3^
0 restraints	Extinction correction: *SHELXL97* (Sheldrick, 2008), Fc^*^=kFc[1+0.001xFc^2^λ^3^/sin(2θ)]^-1/4^
Primary atom site location: structure-invariant direct methods	Extinction coefficient: 0.00170 (18)

### Special details

**Table:**

Geometry. All e.s.d.'s (except the e.s.d. in the dihedral angle between two l.s. planes) are estimated using the full covariance matrix. The cell e.s.d.'s are taken into account individually in the estimation of e.s.d.'s in distances, angles and torsion angles; correlations between e.s.d.'s in cell parameters are only used when they are defined by crystal symmetry. An approximate (isotropic) treatment of cell e.s.d.'s is used for estimating e.s.d.'s involving l.s. planes.
Refinement. Refinement of *F*^2^ against ALL reflections. The weighted *R*-factor *wR* and goodness of fit *S* are based on *F*^2^, conventional *R*-factors *R* are based on *F*, with *F* set to zero for negative *F*^2^. The threshold expression of *F*^2^ > σ(*F*^2^) is used only for calculating *R*-factors(gt) *etc*. and is not relevant to the choice of reflections for refinement. *R*-factors based on *F*^2^ are statistically about twice as large as those based on *F*, and *R*- factors based on ALL data will be even larger.

### Fractional atomic coordinates and isotropic or equivalent isotropic displacement parameters (Å^2^)

**Table:**

	*x*	*y*	*z*	*U*_iso_*/*U*_eq_	
O1	0.15087 (5)	0.3744 (2)	0.18806 (5)	0.0691 (5)	
H1A	0.1686 (9)	0.398 (4)	0.1684 (8)	0.096 (10)*	
O2	0.18374 (5)	0.9439 (2)	−0.02043 (5)	0.0684 (5)	
H2A	0.1652 (9)	0.925 (4)	0.0006 (9)	0.116 (11)*	
O3	0.48619 (5)	0.9288 (2)	0.35466 (5)	0.0684 (5)	
H3A	0.5055 (9)	0.923 (3)	0.3328 (8)	0.099 (9)*	
N1	0.16759 (6)	0.4172 (2)	0.10588 (6)	0.0550 (5)	
N2	0.27218 (8)	0.5702 (4)	0.18410 (8)	0.1010 (8)	
N3	0.16614 (6)	0.9441 (2)	0.06174 (6)	0.0520 (5)	
N4	0.05724 (8)	1.1104 (3)	−0.00802 (8)	0.0945 (8)	
N5	0.50291 (6)	0.9533 (2)	0.27211 (6)	0.0541 (5)	
N6	0.60959 (8)	1.1213 (4)	0.34472 (8)	0.1030 (9)	
C1	0.08638 (7)	0.4080 (3)	0.11845 (7)	0.0515 (6)	
C2	0.10180 (7)	0.3837 (3)	0.16714 (7)	0.0549 (6)	
C3	0.06760 (7)	0.3746 (3)	0.19363 (7)	0.0544 (6)	
C4	0.01780 (7)	0.3800 (3)	0.17000 (7)	0.0594 (6)	
H4A	−0.0056	0.3731	0.1873	0.071*	
C5	0.00084 (7)	0.3952 (3)	0.12154 (7)	0.0555 (6)	
C6	0.03561 (7)	0.4134 (3)	0.09736 (7)	0.0567 (6)	
H6A	0.0254	0.4301	0.0654	0.068*	
C7	−0.05489 (8)	0.3915 (4)	0.09889 (8)	0.0686 (7)	
C8	−0.07643 (9)	0.2160 (5)	0.11300 (10)	0.1213 (12)	
H8A	−0.0712	0.2130	0.1461	0.182*	
H8B	−0.0605	0.1129	0.1031	0.182*	
H8C	−0.1112	0.2115	0.0987	0.182*	
C9	−0.07904 (9)	0.5588 (5)	0.11231 (11)	0.1360 (15)	
H9A	−0.0643	0.6657	0.1028	0.204*	
H9B	−0.0745	0.5608	0.1453	0.204*	
H9C	−0.1137	0.5567	0.0974	0.204*	
C10	−0.06546 (8)	0.3877 (4)	0.04606 (8)	0.0917 (9)	
H10A	−0.0528	0.4968	0.0354	0.138*	
H10B	−0.1005	0.3808	0.0330	0.138*	
H10C	−0.0498	0.2832	0.0366	0.138*	
C11	0.08366 (8)	0.3572 (3)	0.24710 (7)	0.0606 (6)	
C12	0.11144 (8)	0.1771 (4)	0.26034 (8)	0.0839 (8)	
H12A	0.0905	0.0771	0.2470	0.126*	
H12B	0.1207	0.1650	0.2935	0.126*	
H12C	0.1405	0.1764	0.2488	0.126*	
C13	0.11663 (9)	0.5200 (4)	0.26765 (8)	0.0828 (8)	
H13A	0.0988	0.6316	0.2589	0.124*	
H13B	0.1457	0.5198	0.2561	0.124*	
H13C	0.1260	0.5101	0.3008	0.124*	
C14	0.03994 (8)	0.3589 (3)	0.26913 (7)	0.0759 (7)	
H14A	0.0184	0.2586	0.2573	0.114*	
H14B	0.0222	0.4715	0.2618	0.114*	
H14C	0.0518	0.3475	0.3022	0.114*	
C15	0.12062 (8)	0.4301 (3)	0.09044 (7)	0.0550 (6)	
H15A	0.1081	0.4553	0.0591	0.066*	
C16	0.19925 (8)	0.4445 (3)	0.07622 (8)	0.0524 (6)	
C17	0.24714 (8)	0.5059 (3)	0.09644 (8)	0.0567 (6)	
C18	0.28038 (9)	0.5363 (3)	0.07005 (10)	0.0716 (7)	
H18A	0.3121	0.5777	0.0840	0.086*	
C19	0.26641 (10)	0.5051 (4)	0.02291 (10)	0.0794 (8)	
H19A	0.2886	0.5258	0.0048	0.095*	
C20	0.21944 (9)	0.4430 (4)	0.00270 (9)	0.0761 (8)	
H20A	0.2101	0.4226	−0.0291	0.091*	
C21	0.18616 (8)	0.4108 (3)	0.02871 (8)	0.0632 (6)	
H21A	0.1549	0.3664	0.0146	0.076*	
C22	0.26084 (8)	0.5404 (4)	0.14511 (10)	0.0700 (7)	
C23	0.24659 (7)	0.9944 (3)	0.04970 (7)	0.0498 (5)	
C24	0.23205 (7)	0.9758 (3)	0.00082 (7)	0.0524 (6)	
C25	0.26610 (7)	0.9935 (3)	−0.02562 (7)	0.0539 (6)	
C26	0.31490 (7)	1.0267 (3)	−0.00171 (7)	0.0588 (6)	
H26A	0.3383	1.0369	−0.0189	0.071*	
C27	0.33121 (7)	1.0459 (3)	0.04689 (7)	0.0554 (6)	
C28	0.29620 (7)	1.0291 (3)	0.07106 (7)	0.0535 (6)	
H28A	0.3057	1.0411	0.1032	0.064*	
C29	0.25076 (8)	0.9747 (3)	−0.07914 (7)	0.0609 (6)	
C30	0.29447 (8)	1.0043 (4)	−0.10075 (8)	0.0781 (8)	
H30A	0.3198	0.9165	−0.0884	0.117*	
H30B	0.3074	1.1252	−0.0936	0.117*	
H30C	0.2836	0.9898	−0.1338	0.117*	
C31	0.23030 (8)	0.7842 (4)	−0.09238 (8)	0.0809 (8)	
H31A	0.2549	0.6950	−0.0790	0.121*	
H31B	0.2216	0.7721	−0.1255	0.121*	
H31C	0.2015	0.7655	−0.0809	0.121*	
C32	0.21179 (8)	1.1207 (4)	−0.10026 (7)	0.0864 (8)	
H32A	0.1837	1.1074	−0.0875	0.130*	
H32B	0.2017	1.1055	−0.1333	0.130*	
H32C	0.2258	1.2400	−0.0931	0.130*	
C33	0.38584 (7)	1.0851 (3)	0.06904 (8)	0.0656 (7)	
C34	0.40101 (8)	1.2618 (4)	0.04893 (9)	0.1017 (10)	
H34A	0.3957	1.2494	0.0160	0.153*	
H34B	0.4353	1.2857	0.0626	0.153*	
H34C	0.3815	1.3612	0.0556	0.153*	
C35	0.39540 (8)	1.1110 (4)	0.12172 (8)	0.0823 (8)	
H35A	0.3764	1.2125	0.1280	0.124*	
H35B	0.4299	1.1342	0.1347	0.124*	
H35C	0.3859	1.0025	0.1353	0.124*	
C36	0.41687 (8)	0.9228 (4)	0.06066 (9)	0.0991 (10)	
H36A	0.4118	0.9037	0.0279	0.149*	
H36B	0.4072	0.8154	0.0746	0.149*	
H36C	0.4512	0.9474	0.0742	0.149*	
C37	0.21240 (7)	0.9782 (3)	0.07787 (7)	0.0528 (6)	
H37A	0.2242	0.9931	0.1097	0.063*	
C38	0.13442 (7)	0.9239 (3)	0.09128 (7)	0.0498 (5)	
C39	0.14876 (8)	0.8607 (3)	0.13675 (8)	0.0606 (6)	
H39A	0.1817	0.8311	0.1496	0.073*	
C40	0.08411 (8)	0.9640 (3)	0.07310 (8)	0.0543 (6)	
C41	0.05055 (8)	0.9410 (3)	0.09933 (9)	0.0678 (7)	
H41A	0.0174	0.9668	0.0866	0.081*	
C42	0.06576 (9)	0.8799 (3)	0.14438 (9)	0.0725 (7)	
H42A	0.0431	0.8648	0.1622	0.087*	
C43	0.11512 (9)	0.8414 (3)	0.16280 (8)	0.0701 (7)	
H43A	0.1257	0.8017	0.1934	0.084*	
C44	0.06939 (8)	1.0420 (4)	0.02734 (9)	0.0669 (7)	
C45	0.42247 (7)	0.9779 (3)	0.28519 (7)	0.0497 (6)	
C46	0.37210 (7)	1.0054 (3)	0.26459 (7)	0.0524 (6)	
H46A	0.3621	1.0202	0.2326	0.063*	
C47	0.33720 (7)	1.0114 (3)	0.28913 (7)	0.0528 (6)	
C48	0.35431 (7)	0.9892 (3)	0.33760 (7)	0.0575 (6)	
H48A	0.3311	0.9926	0.3551	0.069*	
C49	0.40353 (7)	0.9625 (3)	0.36098 (7)	0.0535 (6)	
C50	0.43758 (7)	0.9546 (3)	0.33395 (7)	0.0524 (6)	
C51	0.28202 (7)	1.0428 (3)	0.26768 (8)	0.0640 (7)	
C52	0.27148 (8)	1.0677 (4)	0.21492 (7)	0.0810 (8)	
H52A	0.2810	0.9593	0.2013	0.121*	
H52B	0.2900	1.1698	0.2081	0.121*	
H52C	0.2369	1.0895	0.2024	0.121*	
C53	0.26556 (8)	1.2169 (4)	0.28790 (9)	0.0998 (10)	
H53A	0.2715	1.2047	0.3209	0.150*	
H53B	0.2309	1.2363	0.2746	0.150*	
H53C	0.2839	1.3191	0.2808	0.150*	
C54	0.25289 (8)	0.8771 (4)	0.27695 (9)	0.0947 (9)	
H54A	0.2591	0.8579	0.3098	0.142*	
H54B	0.2630	0.7712	0.2627	0.142*	
H54C	0.2183	0.8979	0.2641	0.142*	
C55	0.41951 (8)	0.9427 (3)	0.41447 (7)	0.0596 (6)	
C56	0.37603 (8)	0.9617 (4)	0.43647 (7)	0.0748 (7)	
H56A	0.3520	0.8692	0.4243	0.112*	
H56B	0.3613	1.0800	0.4294	0.112*	
H56C	0.3875	0.9478	0.4695	0.112*	
C57	0.45652 (8)	1.0928 (4)	0.43532 (7)	0.0792 (8)	
H57A	0.4663	1.0794	0.4684	0.119*	
H57B	0.4414	1.2101	0.4276	0.119*	
H57C	0.4850	1.0830	0.4230	0.119*	
C58	0.44251 (8)	0.7541 (4)	0.42697 (8)	0.0768 (7)	
H58A	0.4189	0.6616	0.4136	0.115*	
H58B	0.4518	0.7405	0.4601	0.115*	
H58C	0.4712	0.7419	0.4150	0.115*	
C59	0.45650 (7)	0.9795 (3)	0.25691 (7)	0.0524 (6)	
H59A	0.4442	1.0009	0.2253	0.063*	
C60	0.53436 (7)	0.9589 (3)	0.24184 (7)	0.0502 (6)	
C61	0.58307 (8)	1.0150 (3)	0.25981 (8)	0.0542 (6)	
C62	0.61611 (8)	1.0212 (3)	0.23255 (9)	0.0667 (7)	
H62A	0.6485	1.0578	0.2453	0.080*	
C63	0.60124 (9)	0.9730 (3)	0.18625 (9)	0.0738 (7)	
H63A	0.6233	0.9785	0.1675	0.089*	
C64	0.55325 (9)	0.9169 (3)	0.16834 (8)	0.0709 (7)	
H64A	0.5430	0.8843	0.1372	0.085*	
C65	0.52018 (8)	0.9078 (3)	0.19529 (7)	0.0606 (6)	
H65A	0.4881	0.8674	0.1825	0.073*	
C66	0.59803 (8)	1.0731 (4)	0.30739 (9)	0.0685 (7)	

### Atomic displacement parameters (Å^2^)

**Table:**

	*U*^11^	*U*^22^	*U*^33^	*U*^12^	*U*^13^	*U*^23^
O1	0.0411 (9)	0.1099 (14)	0.0502 (10)	0.0016 (9)	0.0002 (8)	0.0064 (9)
O2	0.0390 (9)	0.1115 (14)	0.0492 (10)	−0.0063 (9)	0.0007 (8)	−0.0077 (9)
O3	0.0383 (9)	0.1148 (14)	0.0463 (10)	0.0079 (9)	0.0000 (8)	0.0056 (9)
N1	0.0488 (11)	0.0636 (12)	0.0500 (12)	0.0004 (9)	0.0073 (9)	−0.0037 (9)
N2	0.0710 (15)	0.136 (2)	0.0851 (18)	0.0008 (14)	−0.0003 (14)	−0.0288 (17)
N3	0.0455 (11)	0.0634 (12)	0.0461 (11)	0.0004 (9)	0.0093 (9)	−0.0026 (9)
N4	0.0746 (15)	0.119 (2)	0.0790 (17)	−0.0005 (14)	−0.0018 (13)	0.0166 (15)
N5	0.0441 (10)	0.0683 (13)	0.0480 (11)	0.0040 (9)	0.0081 (9)	−0.0014 (9)
N6	0.0743 (15)	0.140 (2)	0.0859 (18)	0.0056 (14)	0.0030 (14)	−0.0344 (17)
C1	0.0422 (12)	0.0613 (14)	0.0443 (13)	0.0019 (10)	−0.0016 (11)	−0.0056 (11)
C2	0.0391 (12)	0.0692 (15)	0.0487 (14)	0.0021 (11)	−0.0033 (11)	0.0002 (11)
C3	0.0437 (12)	0.0664 (15)	0.0480 (14)	0.0015 (11)	0.0018 (11)	0.0001 (11)
C4	0.0457 (13)	0.0789 (17)	0.0505 (14)	0.0025 (11)	0.0061 (11)	−0.0011 (12)
C5	0.0391 (12)	0.0694 (15)	0.0523 (14)	0.0005 (11)	0.0007 (11)	−0.0026 (12)
C6	0.0464 (13)	0.0689 (15)	0.0465 (13)	0.0017 (11)	−0.0039 (11)	−0.0021 (11)
C7	0.0402 (13)	0.098 (2)	0.0601 (16)	0.0004 (13)	−0.0024 (12)	−0.0027 (14)
C8	0.0559 (16)	0.176 (3)	0.115 (3)	−0.031 (2)	−0.0095 (16)	0.034 (2)
C9	0.0650 (18)	0.191 (4)	0.129 (3)	0.056 (2)	−0.0181 (18)	−0.072 (3)
C10	0.0553 (15)	0.134 (2)	0.0692 (18)	0.0051 (15)	−0.0158 (14)	−0.0109 (17)
C11	0.0547 (14)	0.0782 (17)	0.0439 (13)	0.0018 (12)	0.0029 (11)	0.0064 (12)
C12	0.0742 (16)	0.107 (2)	0.0666 (17)	0.0195 (16)	0.0107 (14)	0.0202 (15)
C13	0.0811 (17)	0.111 (2)	0.0473 (15)	−0.0183 (16)	−0.0004 (14)	−0.0039 (14)
C14	0.0752 (16)	0.098 (2)	0.0534 (15)	0.0074 (14)	0.0147 (13)	0.0070 (14)
C15	0.0538 (14)	0.0620 (15)	0.0441 (14)	−0.0016 (11)	0.0031 (12)	−0.0053 (11)
C16	0.0522 (14)	0.0511 (14)	0.0528 (15)	0.0059 (11)	0.0112 (12)	−0.0008 (11)
C17	0.0484 (14)	0.0576 (15)	0.0607 (16)	0.0048 (11)	0.0073 (13)	−0.0014 (12)
C18	0.0566 (15)	0.0722 (17)	0.087 (2)	0.0037 (13)	0.0189 (16)	0.0056 (15)
C19	0.0749 (19)	0.091 (2)	0.081 (2)	0.0123 (15)	0.0348 (17)	0.0212 (16)
C20	0.0789 (18)	0.091 (2)	0.0594 (17)	0.0123 (16)	0.0199 (16)	0.0071 (14)
C21	0.0587 (15)	0.0723 (16)	0.0552 (15)	0.0024 (12)	0.0080 (13)	−0.0013 (12)
C22	0.0434 (14)	0.0868 (19)	0.0749 (19)	0.0003 (12)	0.0056 (14)	−0.0116 (16)
C23	0.0401 (12)	0.0626 (15)	0.0428 (13)	0.0024 (10)	0.0032 (11)	0.0016 (11)
C24	0.0392 (12)	0.0687 (16)	0.0438 (14)	−0.0008 (11)	0.0002 (11)	−0.0007 (11)
C25	0.0453 (13)	0.0702 (16)	0.0434 (13)	0.0035 (11)	0.0059 (11)	0.0040 (11)
C26	0.0423 (13)	0.0817 (17)	0.0511 (15)	−0.0014 (11)	0.0095 (11)	0.0088 (12)
C27	0.0403 (12)	0.0722 (16)	0.0475 (14)	−0.0010 (11)	−0.0004 (11)	0.0117 (11)
C28	0.0447 (13)	0.0672 (15)	0.0423 (13)	0.0012 (11)	−0.0008 (11)	0.0045 (11)
C29	0.0498 (13)	0.0861 (18)	0.0431 (14)	0.0032 (13)	0.0046 (11)	0.0003 (12)
C30	0.0765 (17)	0.106 (2)	0.0522 (15)	−0.0026 (15)	0.0177 (14)	0.0063 (14)
C31	0.0751 (16)	0.107 (2)	0.0609 (16)	−0.0126 (16)	0.0178 (13)	−0.0162 (15)
C32	0.0784 (17)	0.124 (2)	0.0473 (15)	0.0266 (16)	−0.0018 (13)	0.0121 (15)
C33	0.0394 (12)	0.0897 (19)	0.0598 (16)	−0.0020 (12)	−0.0023 (12)	0.0162 (13)
C34	0.0578 (15)	0.135 (3)	0.099 (2)	−0.0259 (17)	−0.0057 (15)	0.0427 (19)
C35	0.0522 (14)	0.114 (2)	0.0666 (17)	−0.0124 (14)	−0.0108 (13)	0.0019 (15)
C36	0.0494 (15)	0.149 (3)	0.090 (2)	0.0202 (17)	0.0007 (15)	0.0008 (19)
C37	0.0488 (13)	0.0622 (15)	0.0426 (13)	−0.0013 (11)	0.0023 (11)	0.0010 (11)
C38	0.0482 (13)	0.0494 (13)	0.0507 (14)	−0.0024 (10)	0.0107 (11)	−0.0038 (10)
C39	0.0560 (14)	0.0657 (16)	0.0563 (15)	−0.0025 (12)	0.0073 (12)	0.0029 (12)
C40	0.0459 (13)	0.0568 (14)	0.0591 (15)	−0.0012 (11)	0.0110 (12)	−0.0033 (11)
C41	0.0524 (14)	0.0746 (17)	0.0779 (19)	0.0007 (12)	0.0194 (14)	−0.0028 (14)
C42	0.0713 (17)	0.0797 (19)	0.0761 (19)	−0.0118 (14)	0.0363 (15)	−0.0024 (15)
C43	0.0761 (17)	0.0758 (18)	0.0588 (16)	−0.0066 (14)	0.0178 (14)	0.0039 (13)
C44	0.0460 (14)	0.0794 (19)	0.0700 (19)	−0.0003 (12)	0.0048 (14)	−0.0014 (15)
C45	0.0385 (12)	0.0611 (14)	0.0450 (14)	−0.0005 (10)	0.0020 (11)	−0.0043 (10)
C46	0.0424 (12)	0.0703 (16)	0.0382 (12)	0.0002 (11)	−0.0015 (11)	−0.0061 (11)
C47	0.0402 (12)	0.0706 (16)	0.0424 (13)	0.0015 (11)	0.0009 (11)	−0.0087 (11)
C48	0.0418 (13)	0.0805 (17)	0.0491 (14)	−0.0007 (11)	0.0094 (11)	−0.0063 (12)
C49	0.0454 (13)	0.0712 (16)	0.0404 (13)	−0.0007 (11)	0.0042 (11)	−0.0034 (11)
C50	0.0384 (12)	0.0696 (16)	0.0441 (14)	0.0003 (11)	0.0007 (11)	−0.0002 (11)
C51	0.0406 (13)	0.0891 (18)	0.0550 (15)	0.0033 (12)	−0.0013 (11)	−0.0148 (13)
C52	0.0507 (14)	0.118 (2)	0.0624 (17)	0.0142 (14)	−0.0087 (13)	0.0008 (15)
C53	0.0545 (15)	0.132 (3)	0.098 (2)	0.0263 (16)	−0.0081 (14)	−0.0435 (19)
C54	0.0516 (15)	0.143 (3)	0.083 (2)	−0.0193 (16)	0.0044 (14)	−0.0018 (18)
C55	0.0537 (14)	0.0783 (17)	0.0427 (14)	−0.0005 (12)	0.0044 (11)	−0.0006 (12)
C56	0.0736 (16)	0.104 (2)	0.0476 (15)	0.0030 (15)	0.0162 (13)	−0.0052 (13)
C57	0.0760 (16)	0.110 (2)	0.0425 (14)	−0.0182 (15)	−0.0029 (13)	−0.0039 (14)
C58	0.0681 (15)	0.103 (2)	0.0554 (15)	0.0062 (15)	0.0091 (13)	0.0115 (14)
C59	0.0464 (13)	0.0660 (15)	0.0408 (13)	0.0023 (11)	0.0036 (11)	−0.0029 (10)
C60	0.0449 (13)	0.0536 (14)	0.0521 (15)	0.0038 (10)	0.0120 (12)	0.0023 (11)
C61	0.0456 (13)	0.0570 (14)	0.0586 (15)	0.0078 (11)	0.0102 (12)	−0.0001 (11)
C62	0.0484 (14)	0.0741 (17)	0.0768 (19)	0.0041 (12)	0.0143 (14)	0.0062 (14)
C63	0.0645 (17)	0.093 (2)	0.0718 (19)	0.0136 (14)	0.0317 (15)	0.0139 (15)
C64	0.0679 (16)	0.093 (2)	0.0532 (16)	0.0135 (14)	0.0170 (14)	−0.0002 (13)
C65	0.0529 (14)	0.0725 (16)	0.0539 (15)	0.0066 (12)	0.0084 (12)	−0.0024 (12)
C66	0.0461 (14)	0.0821 (19)	0.0739 (19)	0.0069 (12)	0.0088 (14)	−0.0114 (15)

### Geometric parameters (Å, °)

**Table:**

C1—C6	1.402 (3)	C30—H30C	0.9600
C1—C2	1.417 (3)	C31—H31A	0.9600
C1—C15	1.429 (3)	C31—H31B	0.9600
N1—C15	1.282 (2)	C31—H31C	0.9600
N1—C16	1.413 (2)	C32—H32A	0.9600
O1—C2	1.359 (2)	C32—H32B	0.9600
O1—H1A	0.87 (2)	C32—H32C	0.9600
C2—C3	1.386 (3)	C33—C36	1.529 (3)
N2—C22	1.145 (3)	C33—C34	1.531 (3)
O2—C24	1.361 (2)	C33—C35	1.536 (3)
O2—H2A	0.92 (3)	C34—H34A	0.9600
C3—C4	1.395 (2)	C34—H34B	0.9600
C3—C11	1.548 (3)	C34—H34C	0.9600
O3—C50	1.358 (2)	C35—H35A	0.9600
O3—H3A	0.95 (2)	C35—H35B	0.9600
N3—C37	1.287 (2)	C35—H35C	0.9600
N3—C38	1.405 (2)	C36—H36A	0.9600
C4—C5	1.406 (3)	C36—H36B	0.9600
C4—H4A	0.9300	C36—H36C	0.9600
N4—C44	1.138 (3)	C37—H37A	0.9300
C5—C6	1.354 (3)	C38—C39	1.392 (3)
C5—C7	1.536 (3)	C38—C40	1.406 (3)
N5—C59	1.277 (2)	C39—C43	1.369 (3)
N5—C60	1.409 (2)	C39—H39A	0.9300
C6—H6A	0.9300	C40—C41	1.374 (3)
N6—C66	1.134 (3)	C40—C44	1.440 (3)
C7—C9	1.501 (3)	C41—C42	1.377 (3)
C7—C8	1.523 (4)	C41—H41A	0.9300
C7—C10	1.527 (3)	C42—C43	1.381 (3)
C8—H8A	0.9600	C42—H42A	0.9300
C8—H8B	0.9600	C43—H43A	0.9300
C8—H8C	0.9600	C45—C46	1.403 (2)
C9—H9A	0.9600	C45—C50	1.417 (3)
C9—H9B	0.9600	C45—C59	1.421 (3)
C9—H9C	0.9600	C46—C47	1.359 (3)
C10—H10A	0.9600	C46—H46A	0.9300
C10—H10B	0.9600	C47—C48	1.412 (3)
C10—H10C	0.9600	C47—C51	1.532 (3)
C11—C14	1.526 (3)	C48—C49	1.392 (2)
C11—C12	1.532 (3)	C48—H48A	0.9300
C11—C13	1.538 (3)	C49—C50	1.394 (3)
C12—H12A	0.9600	C49—C55	1.550 (3)
C12—H12B	0.9600	C51—C54	1.526 (3)
C12—H12C	0.9600	C51—C53	1.531 (3)
C13—H13A	0.9600	C51—C52	1.535 (3)
C13—H13B	0.9600	C52—H52A	0.9600
C13—H13C	0.9600	C52—H52B	0.9600
C14—H14A	0.9600	C52—H52C	0.9600
C14—H14B	0.9600	C53—H53A	0.9600
C14—H14C	0.9600	C53—H53B	0.9600
C15—H15A	0.9300	C53—H53C	0.9600
C16—C21	1.392 (3)	C54—H54A	0.9600
C16—C17	1.398 (3)	C54—H54B	0.9600
C17—C18	1.378 (3)	C54—H54C	0.9600
C17—C22	1.426 (3)	C55—C56	1.526 (3)
C18—C19	1.379 (3)	C55—C58	1.531 (3)
C18—H18A	0.9300	C55—C57	1.531 (3)
C19—C20	1.378 (3)	C56—H56A	0.9600
C19—H19A	0.9300	C56—H56B	0.9600
C20—C21	1.373 (3)	C56—H56C	0.9600
C20—H20A	0.9300	C57—H57A	0.9600
C21—H21A	0.9300	C57—H57B	0.9600
C23—C28	1.397 (2)	C57—H57C	0.9600
C23—C24	1.416 (3)	C58—H58A	0.9600
C23—C37	1.427 (3)	C58—H58B	0.9600
C24—C25	1.386 (3)	C58—H58C	0.9600
C25—C26	1.393 (3)	C59—H59A	0.9300
C25—C29	1.550 (3)	C60—C65	1.394 (3)
C26—C27	1.411 (3)	C60—C61	1.395 (3)
C26—H26A	0.9300	C61—C62	1.376 (3)
C27—C28	1.359 (3)	C61—C66	1.437 (3)
C27—C33	1.532 (3)	C62—C63	1.383 (3)
C28—H28A	0.9300	C62—H62A	0.9300
C29—C31	1.522 (3)	C63—C64	1.377 (3)
C29—C30	1.533 (3)	C63—H63A	0.9300
C29—C32	1.544 (3)	C64—C65	1.370 (3)
C30—H30A	0.9600	C64—H64A	0.9300
C30—H30B	0.9600	C65—H65A	0.9300
			
C6—C1—C2	118.4 (2)	H32B—C32—H32C	109.5
C6—C1—C15	119.16 (19)	C36—C33—C34	111.7 (2)
C2—C1—C15	122.39 (18)	C36—C33—C27	109.3 (2)
C15—N1—C16	120.80 (19)	C34—C33—C27	109.54 (18)
C2—O1—H1A	111.2 (16)	C36—C33—C35	107.60 (19)
O1—C2—C3	119.90 (19)	C34—C33—C35	107.5 (2)
O1—C2—C1	119.2 (2)	C27—C33—C35	111.24 (19)
C3—C2—C1	120.84 (18)	C33—C34—H34A	109.5
C24—O2—H2A	112.0 (16)	C33—C34—H34B	109.5
C2—C3—C4	117.13 (19)	H34A—C34—H34B	109.5
C2—C3—C11	121.75 (18)	C33—C34—H34C	109.5
C4—C3—C11	121.1 (2)	H34A—C34—H34C	109.5
C50—O3—H3A	112.0 (14)	H34B—C34—H34C	109.5
C37—N3—C38	121.36 (18)	C33—C35—H35A	109.5
C3—C4—C5	123.9 (2)	C33—C35—H35B	109.5
C3—C4—H4A	118.1	H35A—C35—H35B	109.5
C5—C4—H4A	118.1	C33—C35—H35C	109.5
C6—C5—C4	116.83 (18)	H35A—C35—H35C	109.5
C6—C5—C7	123.6 (2)	H35B—C35—H35C	109.5
C4—C5—C7	119.6 (2)	C33—C36—H36A	109.5
C59—N5—C60	120.71 (18)	C33—C36—H36B	109.5
C5—C6—C1	122.7 (2)	H36A—C36—H36B	109.5
C5—C6—H6A	118.6	C33—C36—H36C	109.5
C1—C6—H6A	118.6	H36A—C36—H36C	109.5
C9—C7—C8	112.4 (2)	H36B—C36—H36C	109.5
C9—C7—C10	107.8 (2)	N3—C37—C23	123.80 (19)
C8—C7—C10	106.3 (2)	N3—C37—H37A	118.1
C9—C7—C5	110.1 (2)	C23—C37—H37A	118.1
C8—C7—C5	108.8 (2)	C39—C38—N3	124.82 (19)
C10—C7—C5	111.5 (2)	C39—C38—C40	117.4 (2)
C7—C8—H8A	109.5	N3—C38—C40	117.72 (19)
C7—C8—H8B	109.5	C43—C39—C38	121.0 (2)
H8A—C8—H8B	109.5	C43—C39—H39A	119.5
C7—C8—H8C	109.5	C38—C39—H39A	119.5
H8A—C8—H8C	109.5	C41—C40—C38	121.1 (2)
H8B—C8—H8C	109.5	C41—C40—C44	120.9 (2)
C7—C9—H9A	109.5	C38—C40—C44	117.9 (2)
C7—C9—H9B	109.5	C40—C41—C42	120.4 (2)
H9A—C9—H9B	109.5	C40—C41—H41A	119.8
C7—C9—H9C	109.5	C42—C41—H41A	119.8
H9A—C9—H9C	109.5	C41—C42—C43	119.2 (2)
H9B—C9—H9C	109.5	C41—C42—H42A	120.4
C7—C10—H10A	109.5	C43—C42—H42A	120.4
C7—C10—H10B	109.5	C39—C43—C42	121.0 (2)
H10A—C10—H10B	109.5	C39—C43—H43A	119.5
C7—C10—H10C	109.5	C42—C43—H43A	119.5
H10A—C10—H10C	109.5	N4—C44—C40	177.1 (3)
H10B—C10—H10C	109.5	C46—C45—C50	118.3 (2)
C14—C11—C12	107.80 (19)	C46—C45—C59	119.23 (19)
C14—C11—C13	106.72 (19)	C50—C45—C59	122.45 (18)
C12—C11—C13	110.55 (18)	C47—C46—C45	123.19 (19)
C14—C11—C3	112.63 (17)	C47—C46—H46A	118.4
C12—C11—C3	109.47 (19)	C45—C46—H46A	118.4
C13—C11—C3	109.63 (18)	C46—C47—C48	116.18 (18)
C11—C12—H12A	109.5	C46—C47—C51	124.39 (19)
C11—C12—H12B	109.5	C48—C47—C51	119.4 (2)
H12A—C12—H12B	109.5	C49—C48—C47	124.5 (2)
C11—C12—H12C	109.5	C49—C48—H48A	117.7
H12A—C12—H12C	109.5	C47—C48—H48A	117.7
H12B—C12—H12C	109.5	C48—C49—C50	116.76 (19)
C11—C13—H13A	109.5	C48—C49—C55	121.43 (19)
C11—C13—H13B	109.5	C50—C49—C55	121.82 (18)
H13A—C13—H13B	109.5	O3—C50—C49	119.65 (19)
C11—C13—H13C	109.5	O3—C50—C45	119.30 (19)
H13A—C13—H13C	109.5	C49—C50—C45	121.03 (19)
H13B—C13—H13C	109.5	C54—C51—C53	111.4 (2)
C11—C14—H14A	109.5	C54—C51—C47	109.5 (2)
C11—C14—H14B	109.5	C53—C51—C47	109.39 (18)
H14A—C14—H14B	109.5	C54—C51—C52	107.80 (19)
C11—C14—H14C	109.5	C53—C51—C52	107.7 (2)
H14A—C14—H14C	109.5	C47—C51—C52	111.05 (19)
H14B—C14—H14C	109.5	C51—C52—H52A	109.5
N1—C15—C1	124.0 (2)	C51—C52—H52B	109.5
N1—C15—H15A	118.0	H52A—C52—H52B	109.5
C1—C15—H15A	118.0	C51—C52—H52C	109.5
C21—C16—C17	118.3 (2)	H52A—C52—H52C	109.5
C21—C16—N1	124.3 (2)	H52B—C52—H52C	109.5
C17—C16—N1	117.3 (2)	C51—C53—H53A	109.5
C18—C17—C16	121.1 (2)	C51—C53—H53B	109.5
C18—C17—C22	120.3 (2)	H53A—C53—H53B	109.5
C16—C17—C22	118.6 (2)	C51—C53—H53C	109.5
C17—C18—C19	119.7 (2)	H53A—C53—H53C	109.5
C17—C18—H18A	120.2	H53B—C53—H53C	109.5
C19—C18—H18A	120.2	C51—C54—H54A	109.5
C20—C19—C18	119.7 (2)	C51—C54—H54B	109.5
C20—C19—H19A	120.2	H54A—C54—H54B	109.5
C18—C19—H19A	120.2	C51—C54—H54C	109.5
C21—C20—C19	121.2 (2)	H54A—C54—H54C	109.5
C21—C20—H20A	119.4	H54B—C54—H54C	109.5
C19—C20—H20A	119.4	C56—C55—C58	108.01 (19)
C20—C21—C16	120.0 (2)	C56—C55—C57	106.92 (19)
C20—C21—H21A	120.0	C58—C55—C57	110.48 (19)
C16—C21—H21A	120.0	C56—C55—C49	112.19 (17)
N2—C22—C17	179.1 (3)	C58—C55—C49	109.25 (18)
C28—C23—C24	118.8 (2)	C57—C55—C49	109.96 (18)
C28—C23—C37	118.89 (19)	C55—C56—H56A	109.5
C24—C23—C37	122.30 (18)	C55—C56—H56B	109.5
O2—C24—C25	119.71 (19)	H56A—C56—H56B	109.5
O2—C24—C23	119.35 (19)	C55—C56—H56C	109.5
C25—C24—C23	120.92 (18)	H56A—C56—H56C	109.5
C24—C25—C26	116.80 (19)	H56B—C56—H56C	109.5
C24—C25—C29	121.59 (18)	C55—C57—H57A	109.5
C26—C25—C29	121.6 (2)	C55—C57—H57B	109.5
C25—C26—C27	124.4 (2)	H57A—C57—H57B	109.5
C25—C26—H26A	117.8	C55—C57—H57C	109.5
C27—C26—H26A	117.8	H57A—C57—H57C	109.5
C28—C27—C26	116.37 (18)	H57B—C57—H57C	109.5
C28—C27—C33	124.2 (2)	C55—C58—H58A	109.5
C26—C27—C33	119.4 (2)	C55—C58—H58B	109.5
C27—C28—C23	122.7 (2)	H58A—C58—H58B	109.5
C27—C28—H28A	118.6	C55—C58—H58C	109.5
C23—C28—H28A	118.6	H58A—C58—H58C	109.5
C31—C29—C30	107.98 (19)	H58B—C58—H58C	109.5
C31—C29—C32	110.41 (19)	N5—C59—C45	124.14 (19)
C30—C29—C32	106.64 (19)	N5—C59—H59A	117.9
C31—C29—C25	109.70 (19)	C45—C59—H59A	117.9
C30—C29—C25	112.03 (17)	C65—C60—C61	117.9 (2)
C32—C29—C25	110.02 (19)	C65—C60—N5	124.12 (19)
C29—C30—H30A	109.5	C61—C60—N5	118.0 (2)
C29—C30—H30B	109.5	C62—C61—C60	121.3 (2)
H30A—C30—H30B	109.5	C62—C61—C66	120.0 (2)
C29—C30—H30C	109.5	C60—C61—C66	118.7 (2)
H30A—C30—H30C	109.5	C61—C62—C63	120.1 (2)
H30B—C30—H30C	109.5	C61—C62—H62A	119.9
C29—C31—H31A	109.5	C63—C62—H62A	119.9
C29—C31—H31B	109.5	C64—C63—C62	118.9 (2)
H31A—C31—H31B	109.5	C64—C63—H63A	120.6
C29—C31—H31C	109.5	C62—C63—H63A	120.6
H31A—C31—H31C	109.5	C65—C64—C63	121.5 (2)
H31B—C31—H31C	109.5	C65—C64—H64A	119.2
C29—C32—H32A	109.5	C63—C64—H64A	119.2
C29—C32—H32B	109.5	C64—C65—C60	120.3 (2)
H32A—C32—H32B	109.5	C64—C65—H65A	119.9
C29—C32—H32C	109.5	C60—C65—H65A	119.9
H32A—C32—H32C	109.5	N6—C66—C61	179.0 (3)

### Hydrogen-bond geometry (Å, °)

**Table:**

*D*—H···*A*	*D*—H	H···*A*	*D*···*A*	*D*—H···*A*
O3—H3A···N5	0.95 (2)	1.81 (2)	2.622 (2)	143 (2)
O2—H2A···N3	0.92 (3)	1.82 (3)	2.615 (2)	143 (2)
O1—H1A···N1	0.87 (2)	1.86 (2)	2.623 (2)	145 (2)
